# The Influence of the Patient-Clinician Relationship on Healthcare Outcomes: A Systematic Review and Meta-Analysis of Randomized Controlled Trials

**DOI:** 10.1371/journal.pone.0094207

**Published:** 2014-04-09

**Authors:** John M. Kelley, Gordon Kraft-Todd, Lidia Schapira, Joe Kossowsky, Helen Riess

**Affiliations:** 1 Empathy and Relational Science Program, Psychiatry Department, Massachusetts General Hospital/Harvard Medical School, Boston, Massachusetts, United States of America; 2 Program in Placebo Studies and the Therapeutic Encounter, Beth Israel Deaconess Medical Center/Harvard Medical School, Boston, Massachusetts, United States of America; 3 Psychology Department, Endicott College, Beverly, Massachusetts, United States of America; 4 Department of Medicine, Massachusetts General Hospital, Boston, Massachusetts, United States of America; 5 Department of Anesthesiology, Perioperative and Pain Medicine, Boston Children's Hospital/Harvard Medical School, Boston, Massachusetts, United States of America; 6 Department of Clinical Psychology & Psychotherapy, University of Basel, Basel, Switzerland; Carl von Ossietzky University of Oldenburg, Germany

## Abstract

**Objective:**

To determine whether the patient-clinician relationship has a beneficial effect on either objective or validated subjective healthcare outcomes.

**Design:**

Systematic review and meta-analysis.

**Data Sources:**

Electronic databases EMBASE and MEDLINE and the reference sections of previous reviews.

**Eligibility Criteria for Selecting Studies:**

Included studies were randomized controlled trials (RCTs) in adult patients in which the patient-clinician relationship was systematically manipulated and healthcare outcomes were either objective (e.g., blood pressure) or validated subjective measures (e.g., pain scores). Studies were excluded if the encounter was a routine physical, or a mental health or substance abuse visit; if the outcome was an intermediate outcome such as patient satisfaction or adherence to treatment; if the patient-clinician relationship was manipulated solely by intervening with patients; or if the duration of the clinical encounter was unequal across conditions.

**Results:**

Thirteen RCTs met eligibility criteria. Observed effect sizes for the individual studies ranged from *d* = −.23 to .66. Using a random-effects model, the estimate of the overall effect size was small (*d* = .11), but statistically significant (*p* = .02).

**Conclusions:**

This systematic review and meta-analysis of RCTs suggests that the patient-clinician relationship has a small, but statistically significant effect on healthcare outcomes. Given that relatively few RCTs met our eligibility criteria, and that the majority of these trials were not specifically designed to test the effect of the patient-clinician relationship on healthcare outcomes, we conclude with a call for more research on this important topic.

## Introduction

One of the great challenges of modern medicine is to preserve the finest elements of caregiving in an environment that is increasingly dominated by market forces and routinized practices [1]. Excellent clinicians strive to master not only the theory of disease and treatment, but also to cultivate a therapeutic presence that is commonly believed to improve the experience of patients and to have a beneficial effect on medical outcomes [2], [3]. However, despite this widespread and longstanding belief, the effect of the patient-clinician relationship on healthcare outcomes has rarely been tested in randomized controlled trials. In fact, most empirical studies examining the effect of the patient-clinician relationship on medical outcomes have been observational in nature [4], [5], [6], [7], [8], [9], [10], [11] and therefore cannot assess causality. Nevertheless, these observational studies do suggest that relationship factors may hold important potential to affect health outcomes.

The patient-clinician relationship has both emotional and informational components – what Di Blasi and colleagues have termed emotional care and cognitive care [12]. Emotional care includes mutual trust, empathy, respect, genuineness, acceptance and warmth [13]. Cognitive care includes information gathering, sharing medical information, patient education, and expectation management. Initially, our primary aim was to investigate the emotional component of the patient-clinician relationship. However, most studies of the patient-clinician relationship include both cognitive and emotional care, and consequently, we expanded our focus to include these studies also. We note, however, that studies that do not separately measure emotional care while investigating communication interventions leave unclear which factor – emotional care or cognitive care – is responsible for any beneficial effects. We also note that the boundary between cognitive care such as communications training and emotional care that enhances the patient-clinician relationship is unclear. For example, communications interventions often train clinicians to ask more open-ended questions, to resist interrupting patients, to identify and respond to patient expectations and fears, and to check patients' understanding of the diagnosis and recommended treatment. While these techniques are intended to improve the quality of information exchange, they are also likely to produce richer interpersonal interactions. Indeed, any intervention designed to improve communication – if effectively employed – is also likely to improve the quality of the interpersonal relationship.

Previous reviews have attempted to estimate the magnitude of the effect of relational factors on health outcomes and to discern the relative impact of discrete interventions and contextual factors [12], [14], [15], [16], [17]. Since the last review was published almost a decade ago, and in response to enormous changes in conceptual thinking about how best to restructure the delivery of healthcare services, we undertook an updated systematic review and meta-analysis examining whether the patient-clinician relationship has a beneficial effect on healthcare outcomes.

In contrast to previous reviews, we included in our review only randomized controlled trials (RCTs) that had either objective or validated subjective medical outcomes; and we excluded studies that only examined intermediate outcomes such as patient satisfaction or comprehension of medical advice. Therefore, the current review focuses on the most rigorous sources of evidence to determine whether the relationship between patient and clinician can produce improvements in health. We report here on the thirteen studies that met our selection criteria for study design and methods.

## Methods

We searched the electronic databases EMBASE and MEDLINE from their earliest entries to November 1, 2012. The exact electronic search strategy and a full description are provided in **File S1**. Briefly, the electronic search strategy required that articles: (1) be RCTs written in English and published in a peer-reviewed journal; (2) include in the title or abstract at least one word related to interpersonal skills (e.g., empathy, rapport, relationship, etc.); (3) include in the title or abstract at least one word referring to a clinician (e.g., physician, nurse, dentist, etc.).

For the review by hand, the inclusion criteria were: (1) RCT in adult patients (age ≥18), written in English and published in a peer-reviewed journal; (2) patients were being treated for a specific disorder (i.e., routine physicals were not included); (3) the patient-clinician relationship was systematically manipulated (e.g., improved communication skills, increased empathy, better attention to non-verbal signals, not interrupting, sitting down, making appropriate eye contact, etc.); (4) there was either an objective outcome measure (e.g., blood pressure) or a validated subjective measure (e.g., pain scores). Studies were excluded if: (1) the patient-clinician relationship was manipulated *solely* by intervening with the patients with no manipulation of clinician comportment; (2) the clinicians were mental health professionals; (3) the patients had psychiatric disorders or substance abuse; and (4) clinical encounter time was unequal across conditions. For a detailed description of the inclusion and exclusion criteria, please see **File S2**.

**Figure 1:**
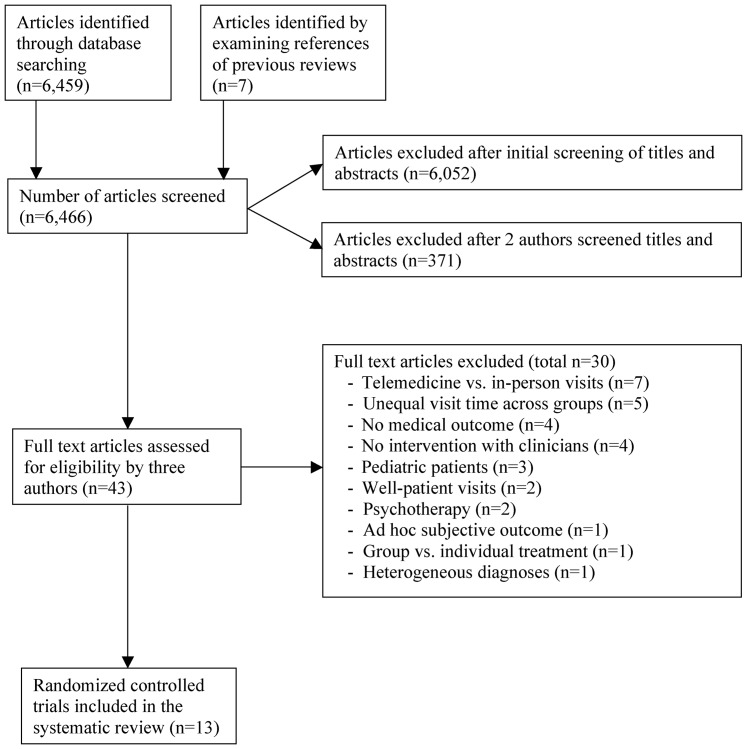
Flow Chart of Study Selection Process.

Our electronic search yielded 6,459 articles. We reviewed the titles and abstracts and eliminated any articles that clearly fell outside our inclusion/exclusion criteria. If there was any doubt, the article was retained for the next level of scrutiny. This process yielded 407 articles. Two authors examined each article's title and abstract more closely and determined that 36 of these should be inspected in depth; again, if there was any doubt, the paper was retained. We also examined the reference sections of previous reviews, and identified an additional 7 articles that potentially met our eligibility criteria. Combined, these processes yielded 43 articles. Three authors then examined the full text of each article and made independent judgments as to whether the article met inclusion/exclusion criteria. Disagreements were resolved by face-to-face discussion, leading to a consensus judgment. Thirteen articles met our inclusion and exclusion criteria. The selection process is illustrated in **Figure 1**.

For the meta-analysis, we computed Cohen's *d* [18], the standardized mean difference in outcomes between the intervention and control groups. As a rule of thumb for the behavioral sciences, Cohen has suggested that *d* = .2 is a small effect, *d* = .5 is medium, and *d* = .8 is large. If a primary outcome was specified, we used that outcome. If more than one primary outcome was specified, we averaged across those outcomes. And if no primary outcome was specified, we averaged across all reported outcomes. Given the heterogeneity of clinicians studied, interventions employed, and outcome measures assessed, we chose to use a random-effects model to summarize across studies. A random-effects model assumes that the true intervention effect size varies depending on characteristics of the population studied or intervention employed. A random-effects model is more conservative than a fixed-effects model because it produces a wider confidence interval for the summary effect size. Standardized mean differences estimates were pooled, using the inverse of their variances as weights [19].

To assess heterogeneity between studies, Q-statistics were calculated. A statistically significant Q indicates a heterogeneous distribution of effect sizes between studies, meaning that systematic differences, possibly influencing the results, are present [20]. Further, we calculated tau-squared, a point estimate of the among-study variance of true effects [21]. In addition, the degree of inconsistency was quantified by the I^2^ statistic, which measures the percentage of variation across studies that is due to heterogeneity rather than chance [22]. An I^2^ value of 25% is categorized as low heterogeneity, 50% as moderate, and 75% as high [22].

Additional sensitivity analyses explored the effects of various possible sources of artifact or bias on the results. First, we assessed the presence of publication bias visually by funnel plot [23] and formally by its direct statistical analogue, Begg's adjusted-rank correlation test [24]. We also used Rosenthal's fail-safe N method [25] to determine the number of unpublished or un-retrieved null studies that would need to exist for the combined effect size to no longer be statistically significant. Sensitivity to the estimate of publication bias was assessed by the trim-and-fill method [26]. Two independent raters (JMK and JK) used the Cochrane Collaboration's tool for assessing the risk of bias [27].

## Results

The thirteen articles that met our inclusion and exclusion criteria are summarized in **Table 1**. All studies were published after 1997. Eight studies were conducted in Europe, four in the United States, and one in Australia. Three trials included patients with diabetes, two included patients with osteoarthritis; no other disorder was represented more than once. The median patient sample size was 279 (range: 85 to 7,557). The median clinician sample size was 39 (range: 3 to 180; two studies did not report clinician sample size). Nine papers studied physicians, two studied a mix of physicians and other medical personnel, one studied acupuncturists, and one studied nurses.

**Table 1:** Characteristics of Studies Included in the Systematic Review.

Author	Year	Country	Patients	N	Clinicians	N	Outcome Meaures
Aiarzaguena	2007	Spain	Somatic complaints	156	MD	39	Health-related quality of life
Bieber	2006	Germany	Fibromyalgia	85	MD	10	Pain, depression, functioning,
Bolognesi	2006	Italy	Obesity	96	MD	8	Weight loss
Cals	2009	Netherlands	Lower resp. infection	431	MD	40	Re-consultation rate
Chassany	2006	France	Osteoarthritis	818	MD	180	Pain relief
Christian	2008	USA	Diabetes	310	MD	NR	Weight loss
Cleland	2007	Scotland	Asthma	629	RN	NR	Asthma quality of life
Cooper	2001	USA	Hypertension	279	MD	41	Blood pressure
Girgis	2009	Australia	Oncology	375	MD	29	Psychosocial (e.g., anxiety, depression)
Kinmonth	1998	UK	Diabetes	250	MD and RN	107	Blood pressure, serum levels, psychosocial
Sequist	2010	USA	Diabetes	7557	MD, NP, and PA	124	Blood pressure, serum levels
White	2011	UK	Osteoarthritis	279	Acupuncturists	3	Osteoarthritis pain
Williams	2001	USA	Smoking	249	MD	27	Smoking quit rate

**Note**s: MD = physician, RN = nurse, NP = nurse practitioner, PA = physician's assistant, NR = not reported.

**Figure 2:**
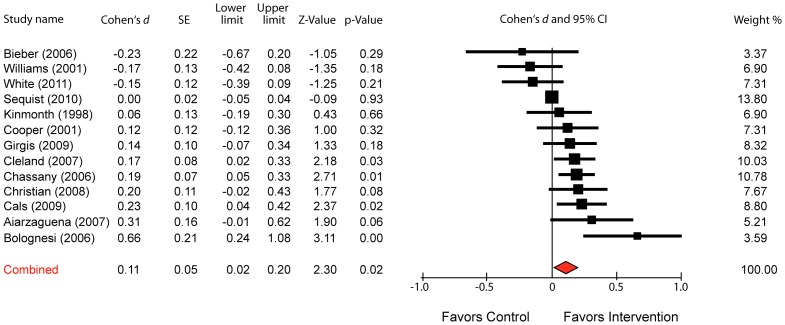
Forest Plot of Cohen's *d* for the Effect of the Patient-Clinician Relationship on Healthcare Outcomes.

To compute effect sizes, we used Cohen's *d*, the standardized mean difference between groups. As shown in the forest plot in **Figure 2**, observed effect sizes for the individual studies ranged from *d* = −.23 to .66; and using a random-effects model, the estimate of the combined effect size was *d* = .11. Even though the overall effect was modest in size, it was statistically significant (p = .02). The studies showed low between-study heterogeneity (*Q* = 14.96, df = 12, *p* = .244, *I* ^2^ = 19.77, Tau-squared = .015). There was no evident publication bias in a funnel plot, the result of Begg's test was not significant (*p* = .27) and the fail-safe N indicated that 32 unpublished or un-retrieved null studies would be needed for the findings to no longer be statistically significant. The trim and fill method did not lead to any adjustment of the standardized mean difference.

**Figure 3:**
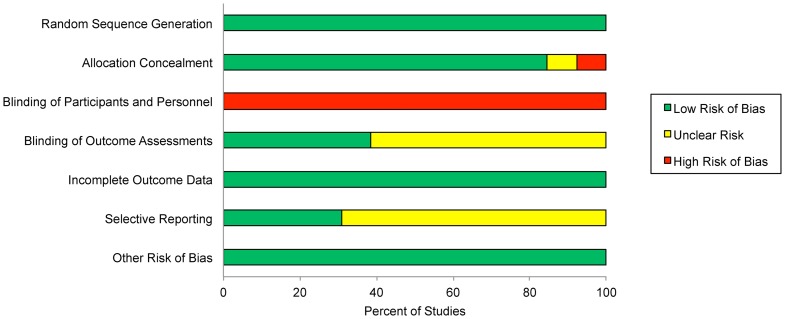
Risk of Bias Assessment.

**Table 2** displays an assessment of the risk of bias for each study using a tool developed by the Cochrane Collaboration [27]. The risk of bias across the included studies was generally low and is summarized in **Figure 3**. The largest potential source of bias arises from the fact that it is impossible to blind treating clinicians to their allocation assignment in these sorts of studies. One might expect that lack of blinding of the treating clinicians would tend to favor the intervention over the control. However, it is possible that elimination of this potential bias could favor the control over the intervention and change our conclusion that there is a statistically significant effect for the influence of the therapeutic relationship on healthcare outcomes.

**Table 2:** Assessment of Risk of Bias.

Author	Year	Random Sequence Generation	Allocation Concealment	Blinding of Participants and Personnel	Blinding of Outcome Assessment	Incomplete Outcome Data	Selective Reporting	Other Risks of Bias
Aiarzaguena	2007	Low risk	Low risk	High risk	Unclear	Low risk	Unclear	Low risk
Bieber	2006	Low risk	Unclear	High risk	Unclear	Low risk	Unclear	Low risk
Bolognesi	2006	Low risk	High risk	High risk	Low risk	Low risk	Unclear	Low risk
Cals	2009	Low risk	Low risk	High risk	Low risk	Low risk	Low	Low risk
Chassany	2006	Low risk	Low risk	High risk	Unclear	Low risk	Unclear	Low risk
Christian	2008	Low risk	Low risk	High risk	Low risk	Low risk	Unclear	Low risk
Cleland	2007	Low risk	Low risk	High risk	Unclear	Low risk	Unclear	Low risk
Cooper	2001	Low risk	Low risk	High risk	Low risk	Low risk	Low	Low risk
Girgis	2009	Low risk	Low risk	High risk	Unclear	Low risk	Unclear	Low risk
Kinmonth	1998	Low risk	Low risk	High risk	Unclear	Low risk	Unclear	Low risk
Sequist	2010	Low risk	Low risk	High risk	Low risk	Low risk	Low	Low risk
White	2011	Low risk	Low risk	High risk	Unclear	Low risk	Low	Low risk
Williams	2001	Low risk	Low risk	High risk	Unclear	Low risk	Unclear	Low risk

Three studies [28], [29], [30] used a within-clinicians design such that each clinician saw patients in *both* the intervention and control conditions. All other studies used a between-clinicians design such that clinicians saw patients in *either* the intervention or the control condition. Four of the studies with a between-clinicians design used cluster randomization, such that entire practices were randomized to either the intervention or the control condition [31], [32], [33], [34]. Cals [31] had 20 clusters and a total of 431 patients; Cleland [32] had 13 clusters and 629 patients; Kinmonth [33] had 41 clusters and 250 patients; and Sequist [34] had 31 clusters and 7,557 patients. All four studies adjusted for clustering in their statistical analyses. Intracluster correlation coefficients were generally low (all below .06, but Sequist [34] did not report the coefficient). All other studies randomized clinicians at the individual level.

The interventions used to alter the patient-clinician relationship varied considerably. Six trials [31], [32], [35], [36], [37], [38] used interventions designed to improve communication skills. Three trials [28], [30], [39] used some form of motivational interviewing based on the stages of change model [40]. One trial used shared decision making [41], one used patient-centered care [33], one used empathic care [29], and one used cultural competency training [34].

Control conditions also varied to some degree. Ten trials used a treatment as usual control [28], [31], [32], [33], [34], [36], [37], [38], [39], [41], [42]; one trial used the Goldberg reattribution technique as a control [35]; one asked clinicians to be less empathic and to minimize any talking with patients [29]; and one asked clinicians to act in a controlling manner, emphasizing clinician power and minimizing patient autonomy [30].

Eight trials augmented the relationship intervention (but not the control) with a variety of additional elements aimed at improving healthcare outcomes. Of these eight trials, three provided patients with written materials to encourage healthy behavior [32], [33], [36]; two assessed patients prior to their appointments and provided feedback to either the clinician or the patient [28], [39]; one gave patients coaching on communication skills prior to healthcare visits [37]; one provided a physical explanation for somatic patients' symptoms [35]; and one gave physicians monthly performance reports [34].

We consider these eight trials “impure” tests of the effect of the patient-clinician relationship because the relationship manipulation is confounded with the additional elements added to the intervention. Therefore, it is not possible to determine whether the results are due to the relationship or to the additional elements. Only five trials [29], [30], [31], [38], [41] provided a “pure” test of the relationship intervention without the addition of other factors thought to improve healthcare outcomes.

## Discussion

Thirteen RCTs met eligibility criteria. Using a random-effects model, our meta-analysis indicated that the patient-clinician relationship has a small (*d* = .11), but statistically significant (*p* = .02) effect on healthcare outcomes. Although the current study estimates that the effect size for the influence of the clinical relationship on healthcare outcomes is small, it's important to note that effect sizes for many important variables affecting health are of similarly small magnitude. For example, the effect size for aspirin in reducing myocardial infarction over five years is only *d* = .06; and the effect size for the influence of smoking on male mortality over 8 years is only *d* = .08 [43]. Effect sizes in medicine are often small because there are many factors that influence health outcomes (e.g., severity of disease, ancillary treatments, co-morbidity, psychosocial stressors, natural course of illness, regression to the mean, etc.). For these reasons, the therapeutic relationship – like many other important variables – may only account for a small fraction of the variance in health outcomes.

There are several other factors that may have attenuated the effect size for the influence of the clinical relationship on health outcomes. First, most of the reviewed trials were not explicitly designed to test the patient-clinician relationship, but rather investigated the therapeutic relationship as one component in a package of interventions. Such studies may have paid insufficient attention to the healthcare relationship, thus limiting effectiveness. Second, there are many ways to implement changes to the patient-clinician relationship, and it is unclear which is most effective in general, or if one intervention can meet the needs of all patients. Studies that restrict the clinician's flexibility by imposing a single technique or communication style may lead to inferior outcomes. Third, it is possible that there was insufficient contact between clinicians and patients, which could also reduce effect sizes.

An anonymous reviewer commented on the fact that we excluded studies that manipulated the healthcare relationship *solely* from the patient side. The reviewer noted the irony of focusing principally on only one side of a relationship that necessarily involves two parties. We agree that interventions that focus on patients may be effective; however, from a purely practical standpoint, there is far more opportunity to implement substantial interpersonal trainings for healthcare professionals than there is to do the same for patients. For example, any intervention aimed at patients would need to be voluntary, simple, and brief. Moreover, to make an impact on healthcare outcomes in the population, training for patients would need to be delivered to all patients with the targeted disorder – a very tall order indeed, given that the ratio of patients to clinicians is extremely large. In contrast, there is ample opportunity for clinicians to receive interpersonal training during their professional and continuing education.

There have been several previous reviews focusing on the effect of the therapeutic relationship in healthcare [12], [14], [15], [16], [17]. The current study differs from these previous reviews in that we excluded observational studies, as well as studies that used intermediate outcomes such as patient satisfaction, adherence to treatment, or patient comprehension of medical advice. Although these intermediate variables are likely to be important mediators of health outcomes, showing change on intermediate variables is insufficient for demonstrating efficacy on health outcomes (e.g., a patient might be highly satisfied with treatment, but not show improved health). Thus, in contrast to previous reviews, the eligibility criteria of the current systematic review required a higher standard of evidence, which may explain why our findings were less positive than previous reviews.

Di Blasi and colleagues [12] conducted a systematic review in 2001 that bears some similarity to the present study. Both reviews included only randomized controlled trials involving patients with physical illnesses in which there was a manipulation of some aspect of the patient–clinician relationship. Both also excluded studies of substance abusers or psychiatric patients. However, the eligibility criteria for the current study differed from Di Blasi in several important ways that made our criteria stricter. Most importantly, we required that there be either an objective healthcare outcome or a validated subjective healthcare outcome. Unlike Di Blasi, we excluded studies that used intermediate outcomes such as satisfaction with treatment, adherence to treatment or screening recommendations, improvements in patient comprehension of medical advice, or changes in clinician behavior (e.g., reductions in antibiotic prescription rates). Thus, our review focused exclusively on RCTs with medical outcomes. We also excluded studies that solely used informational interventions (e.g., training practitioners to adhere to established clinical care guidelines) and studies that manipulated the patient-clinician relationship solely from the patient side. These differences from Di Blasi arose from the fact that our primary interest was to determine whether training clinicians to improve interactions with patients could affect medical outcomes. These stricter criteria resulted in fewer included studies.

This meta-analysis has several limitations. First, study of the patient-clinician relationship is a complex undertaking and definitions and naming conventions are heterogeneous. Consequently, despite a rigorous search process, we may have missed some studies that would have met our eligibility criteria. Second, this review was limited to studies published in peer-reviewed journals, and unpublished studies were not included. Third, this study only included reports written in English, and it is possible that studies written in other languages might have met our other inclusion criteria. Fourth, because this study was restricted to randomized controlled trials, our findings are relevant to efficacy, but may not accurately gauge effectiveness in routine clinical care. Fifth, this review excluded studies of children, substance abusers, patients with psychiatric conditions, and studies of interventions conducted by mental health professionals; and therefore, our findings cannot be generalized to these populations.

In summary, thirteen RCTs met the eligibility criteria of this systematic review. Using a random-effects model, meta-analysis suggests that the patient-clinician relationship has a small, but statistically significant effect on healthcare outcomes. Future rigorously designed RCTs with large sample sizes will be essential to more fully explore the impact of the patient-clinician relationship on medical outcomes.

## Supporting Information

Checklist S1 PRISMA Checklist.
(DOC)

File S1 Electronic Search Strategy.
(DOCX)

File S2 Inclusion/Exclusion Criteria.
(DOCX)
