# Correction: The Influence of the Patient-Clinician Relationship on Healthcare Outcomes: A Systematic Review and Meta-Analysis of Randomized Controlled Trials

**DOI:** 10.1371/journal.pone.0101191

**Published:** 2014-06-18

**Authors:** The PLOS ONE Staff

The affiliation for first author John M. Kelley is incorrect. He is affiliated with #1, #2, and #3, which are listed below.

John M. Kelley^1,2,3^

1 Empathy and Relational Science Program, Psychiatry Department, Massachusetts General Hospital/Harvard Medical School, Boston, Massachusetts, United States of America

2 Program in Placebo Studies and the Therapeutic Encounter, Beth Israel Deaconess Medical Center/Harvard Medical School, Boston, Massachusetts, United States of America

3 Psychology Department, Endicott College, Beverly, Massachusetts, United States of America

The following information is also missing from the Funding section: This study was supported by a grant from the Endicott College Faculty Research Fund.
