# Supplementary material for: The Influence of the Patient-Clinician Relationship on Healthcare Outcomes: A Systematic Review and Meta-Analysis of Randomized Controlled Trials
Source: PLoS One. 2014 Apr 9;9(4):e94207. doi: 10.1371/journal.pone.0094207 (PMC3981763; doi:10.1371/journal.pone.0094207)
Supplement: File S1 — Electronic Search Strategy. (DOCX) [file pone.0094207.s002.docx]

**Supporting File S1**

**Electronic Search Strategy**

The following is the electronic search terms we used in EMBASE and MEDLINE:

empath* OR communication* OR relationship* OR interpersonal OR rapport OR ('patient'/exp AND cent*red) AND (caregiver* OR healer* OR therapist* OR resident* OR surgeon* OR dentist* OR 'midwife'/exp OR 'midwives'/exp OR acupuncturist* OR gp* OR physician* OR nurse* OR doctor* OR practitioner* OR clinician* OR professional* OR clinician*) AND [humans]/lim AND [english]/lim AND [randomized controlled trial]/lim

This search strategy can be described more clearly in English as follows. The three numbered items below were combined using the AND command in Boolean logic.

1. Search for terms in the title or abstract related to an intervention to alter the patient-doctor relationship:
   1. Communication OR
   2. Empath* (captures: empathy, empathic, empathetic, etc.) OR
   3. Relationship* (captures: relationship and relationships) OR
   4. Interpersonal OR
   5. Rapport OR
   6. Patient AND Cent*red (captures: patient-centered, patient-centred, patient centered, patient centred)
2. AND search for terms in the title or abstract indicating that a health care professional was involved:
   1. Physician* (asterisks capture the plural of each term in this section)
   2. Doctor*
   3. Surgeon*
   4. Dentist*
   5. GP*
   6. Resident*
   7. Nurse*
   8. Practitioner*
   9. Clinician*
   10. Professional*
   11. Clinician*
   12. Healer*
   13. Caregiver*
   14. Therapist*
   15. Acupuncturist*
   16. Midwife
   17. Midwives
3. AND limit search to:
   1. The article is in English AND
   2. The study involves humans AND
   3. The study was an RCT
