# Supplementary material for: The Influence of the Patient-Clinician Relationship on Healthcare Outcomes: A Systematic Review and Meta-Analysis of Randomized Controlled Trials
Source: PLoS One. 2014 Apr 9;9(4):e94207. doi: 10.1371/journal.pone.0094207 (PMC3981763; doi:10.1371/journal.pone.0094207)
Supplement: File S2 — Inclusion/Exclusion Criteria. (DOCX) [file pone.0094207.s003.docx]

**Supporting File S2**

**Inclusion/Exclusion Criteria**

1. Must be an RCT written in English and published in a peer-reviewed journal
2. Patients
   1. Adults (age ≥18). Note: children and adolescents are excluded because the patient-clinician relationship often involves parents or guardians as well as the minor patient.
   2. Exclude well-patient visits, **unless** there’s a targeted disorder (e.g., include regular, non-specific check-up in which the practitioner identifies a specific problem, such as smoking, obesity, or hypertension).
   3. Exclude Substance Abuse (but include smoking)
   4. Exclude psychiatric disorders (**except** as noted below)
   5. Include somatization, fibromyalgia, chronic fatigue, IBS, and other disorders that might have a psychological component but are not purely psychiatric disorders
   6. Include patients with anxiety, depression, distress, fatigue, sexual dysfunction if they are secondary to a medical disorder
3. Healthcare clinician
   1. Exclude family or friends or other laypeople who care for the patient
   2. Exclude mental health workers (e.g., psychologist, psychiatrist, psychiatric nurse)
   3. Include all other paid healthcare clinicians (e.g,. doctor, dentist, nurse, acupuncturist, etc.)
   4. Exclude the addition of an outside practitioner to the patient-clinician relationship (e.g., comparing the addition of a trained nurse to facilitate communication versus treatment as usual), **unless** the outside person is added to both experimental and control conditions, and the nature of the patient-practitioner relationship is varied systematically as defined in #4 below.
4. The intervention must change the patient-clinician interaction (e.g., improved communication skills, increased empathy, better attention to non-verbal signals, not interrupting, sitting down, making eye contact, using techniques derived from psychotherapy such as cognitive restructuring or motivational interviewing, etc.)
   1. Exclude if the patient-clinician relationship was manipulated *solely* by intervening with the patients with no manipulation of clinician comportment Exclude if the intervention is purely informational or procedural.
   2. Include if the intervention is informational and/or procedural **but also** includes changes in the quality of the interaction as defined above.
   3. Include if the intervention is derived from psychology (e.g., CBT, motivational interviewing) but only if the intervention is delivered by a healthcare clinician who is **not** a mental health professional.
   4. Exclude if the “intervention” simply varies how the patient-clinician interaction occurs by contrasting in-person vs. phone; or in-person vs. Skype, etc.
   5. Exclude if time across treatment groups is not equal.
5. Healthcare outcome
   1. Objective (e.g., blood pressure)
   2. Validated subjective measure (e.g., SF-36)
   3. Include
      1. Weight loss
      2. Smoking reduction
      3. Exercise frequency
      4. Days off work, days with restrictions, days affected with symptoms
      5. Healthcare utilization, ED visits, readmissions, unplanned admissions, re-consultation rates
   4. Exclude
      1. Antibiotic prescription rates
      2. Colonoscopy screening rates
6. Control Condition
   1. The only difference between the control and experimental conditions is in the nature of the patient-clinician interaction (e.g., for the experimental group but not the control, there is improved communication skills, increased empathy, better attention to non-verbal signals, not interrupting, sitting down, making eye contact, active listening skills, using techniques derived from psychotherapy such as cognitive restructuring or motivational interviewing, etc.).
   2. The control condition includes an equal amount of time with the clinician, either as treatment as usual (TAU) or some other control intervention (e.g., information).
   3. Include control conditions that are “intent-to-fail” (e.g., the control condition requires that clinicians be less empathic and less engaged than they would ordinarily be).
